# Glycopolymer and Poly(β-amino ester)-Based Amphiphilic Block Copolymer as a Drug Carrier

**DOI:** 10.1021/acs.biomac.2c01076

**Published:** 2022-11-01

**Authors:** Elif L. Sahkulubey Kahveci, Muhammet U. Kahveci, Asuman Celebi, Timucin Avsar, Serap Derman

**Affiliations:** † Faculty of Chemical and Metallurgical Engineering, Department of Bioengineering, Yildiz Technical University, Davutpasa Campus, Esenler, 34210Istanbul, Turkey; ‡ Faculty of Science and Letters, Department of Chemistry, Istanbul Technical University, Maslak, Sariyer, 34467Istanbul, Turkey; § Department of Medical Biology, School of Medicine, Bahcesehir University, Goztepe, 34734Istanbul, Turkey

## Abstract

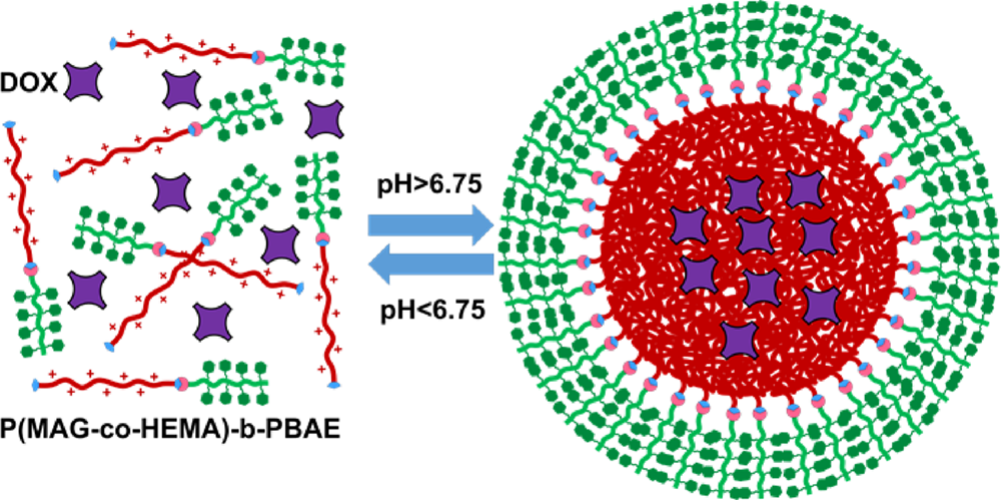

Glycopolymers are synthetic macromolecules having pendant sugar moieties and widely utilized to target cancer cells. They are usually considered as a hydrophilic segment of amphiphilic block copolymers to fabricate micelles as drug carriers. A novel amphiphilic block copolymer, namely, poly(2-deoxy-2-methacrylamido-d-glucose-*co*-2-hydroxyethyl methacrylate)-*b*-poly(β-amino ester) [P(MAG-*co*-HEMA)-*b*-PBAE], with active cancer cell targeting potential and pH responsivity was prepared. Tetrazine end functional P(MAG-*co*-HEMA) and norbornene end functional PBAE blocks were separately synthesized through reversible addition fragmentation chain transfer polymerization and Michael addition-based poly-condensation, respectively, and followed by end-group transformation. Then, inverse electron demand Diels Alder reaction between the tetrazine and the norbornene groups was performed by simply mixing to obtain the amphiphilic block copolymer. After characterization of the block copolymer in terms of chemical structure, pH responsivity, and drug loading/releasing, pH-responsive micelles were obtained with or without doxorubicin (DOX), a model anticancer drug. The micelles exhibited a sharp protonated/deprotonated transition on tertiary amine groups around pH 6.75 and the pH-specific release of DOX below this value. Eventually, the drug delivery potential was evaluated by cytotoxicity assays on both the noncancerous human umbilical vein endothelial cell (HUVEC) cell line and glioblastoma cell line, U87-MG. While the DOX-loaded polymeric micelles were not toxic in noncancerous HUVEC cells, being toxic only to the cancer cells indicates that it is a potential specific cell targeting strategy in the treatment of cancer.

## Introduction

Advanced drug delivery systems are described as integrated materials or devices to deliver therapeutic agents in a site-directed fashion and/or to tune release kinetics.^1^ They offer many advantages including enhanced drug stability and solubility, facilitated passage across biological barriers, prolonged circulation times leading to improved bioavailability, efficacy, and safety.^2,3^ In addition, these systems allow targeted delivery resulting in the accumulation of therapeutics at the diseased area, and also controlled kinetics of the release.^4^ Therefore, advanced drug delivery systems have been recently considered as an important element of treatment of diseases in terms of maximizing efficacy of therapeutics and minimizing their side effects.^1^ In recent years, tremendous efforts have been focused on the development of drug delivery systems for many diseases, especially cancer. Stimuli responsive polymeric micelles as drug delivery systems have been used for the controlled release of drugs into the action of site only in response to environmental or physical stimuli, such as low pH, temperature, enzyme, sound, redox, or light.^5−7^ pH-sensitive polymers provide a specific opportunity for the targeted treatment of cancer, since the increased glucose metabolism of cancer cells causes accumulation of H^+^ ions and, as a result, lowers the pH in the tumor microenvironment ranging from 5.7 to 7.8.^8−11^ Furthermore, subcellular compartments such as lysosomes have much lower pH, 5.0–5.5.^12^ As normal tissues have a pH of 7.4, the pH difference between normal tissues and tumor tissue/lysosome has allowed the development of several pH-sensitive polymeric drug delivery materials for cancer treatment.^11,13−20^

Poly(β-amino ester) (PBAE) is one of the pH-responsive polymers containing tertiary amine groups with a p*K*_b_ value around 6.5. As pH decreases below the p*K*_b_, the tertiary amines are protonated, and the polymer becomes a cationic polymer with high solubility in aqueous solutions. This cationic polymer can readily react with negatively charged molecules such as DNA and RNA and form a complex called polyplex. Therefore, PBAEs have been widely utilized in gene delivery since they were introduced as noncytotoxic and biodegradable DNA vectors by Langer and co-workers in 2000.^21−23^ Because of the protonation and deprotonation of the tertiary amine group, PBAEs have been considered as promising pH-sensitive drug delivery materials for tumor targeting.^24−27^ For instance, several PBAEs in combination with poly(ethylene glycol) (PEG) as the hydrophilic segment have been utilized as drug carriers in the form of hydrogels,^28,29^ micelles,^30−33^ blends,^34,35^ etc. Even, PBAE-based nanoparticles were developed for the co-delivery of anticancer chemotherapeutics (i.e., doxorubicin (DOX)) and a RNA molecule or proapoptotic peptide to develop a system to treat drug resistant cancer more efficiently.^36^ In another study, D-α-tocopheryl PEG succinate incorporated PBAE was fabricated for overcoming multidrug resistance.^37^ Therefore, PBAE is an elegant candidate to develop a pH-responsive drug carrier with different properties.

In addition to variation in pH, another important difference of the cancer cells is the overexpression of various membrane proteins such as growth factor receptors (e.g., epidermal growth factor receptors), hormone receptors, transferrin receptors,^38^ folate receptors,^39^ lectins,^40^ and glucose transporters^41^ (GLUTs) which are responsible in growth, differentiation, and high metabolism of the cancer cells.^4,42^ Therefore, these receptors are widely employed in diagnostic tools and drug delivery systems that specifically target cancer cells.^42,43^ One of these overexpressed proteins is GLUTs that take up glucose more effectively, because cancer cells consume a much higher amount of sugar compared to healthy cells.^41,44^ In addition, a polysaccharide binding membrane glycoprotein involved in several cell–cell interactions, namely, CD44, is overexpressed in tumor cells. Therefore, using sugar moieties as ligands of either GLUTs or CD44 to actively target cancer cells is becoming one of the important strategies in cancer therapy.^45−47^ Glycopolymers are synthetic macromolecules having pendant sugar moieties and widely used to target cancer cells.^40,48−50^ They are usually utilized as the hydrophilic segment of amphiphilic block copolymers to fabricate micelles as drug carriers.^51,52^ One of these glycopolymers is poly(2-deoxy-2-methacrylamido-d-glucose) (PMAG) mostly obtained by reversible addition fragmentation chain transfer (RAFT) polymerization and has been extensively studied in delivery applications. Since PMAG is hydrophilic, it is usually combined with hydrophobic segments including poly(l-lysine-*co*-*L*-phenylalanine),^53^ poly[(*N*-(2-aminoethyl) methacrylamide],^54^ poly[*N*-[3-(*N,N*-dimethylamino) propyl] methacrylamide],^55^ and poly(*O*-cholesteryl methacrylate)^56^ to fabricate core–shell micelles. Such polymeric micelles are useful in both passive targeting due to their sizes (enhanced permeation and retention effect)^57^ and active targeting via glucose groups^40^ leading to decreased systemic toxicity and side effects.

To the best of our knowledge, glycopolymer and PBAE-based block copolymers have not been reported; thus, a novel pH-responsive amphiphilic block copolymer, namely, PMAG-*co*-2-hydroxyethyl methacrylate)-*b*-PBAE) [P(MAG-*co*-HEMA)-*b*-PBAE], with active cancer cell targeting potential was synthesized for the first time. Tetrazine end functional P(MAG-*co*-HEMA) and norbornene end functional PBAE blocks were individually synthesized through RAFT polymerization and Michael addition type poly-condensation, respectively, and subsequent end-group transformations. Then, the amphiphilic block copolymer was obtained through an inverse electron demand Diels Alder (IEDDA) reaction between the tetrazine and the norbornene groups by simply mixing. After characterization of the block copolymer, pH responsivity and drug loading/releasing of the micellar structures produced from the block copolymer were evaluated with DOX as a model anticancer drug. Eventually, anticancer drug delivery potential was examined via cell viability assays for both the noncancerous human umbilical vein endothelial cell (HUVEC) cell line and glioblastoma cell line U87-MG.

## Experimental Section

### Materials

1,4-Butanediol diacrylate (BDA) (90%, Sigma Aldrich), 5-amino-1-pentanol (AP) (95%, Sigma Aldrich), 5-norbornene-2-methylamine (mixture of isomers, TCL), d-(+)-glucosamine hydrochloride (Sigma Aldrich), methacryloyl chloride (97%, Sigma Aldrich, contains 200 ppm monomethyl ether hydroquinone as a stabilizer), potassium carbonate (Alfa Aesar),HEMA (Sigma Aldrich), 4-cyano-4-[(dodecylsulfanylthiocarbonyl)sulfanyl]pentanoic acid (97%, HPLC, Sigma Aldrich), azobisisobutyronitrile (AIBN) (98%, Sigma Aldrich), *N*-(3-dimethylaminopropyl)-*N′-*ethylcarbodiimide hydrochloride (EDC) (Sigma Aldrich), *N*-hydroxysuccinimide (NHS) (Merck), triethyl amine (Et_3_N) (Sigma Aldrich), and all other chemicals were of analytical grade, obtained from commercial suppliers, and used without further purification unless otherwise specified. Tetrazine amine (Tz-NH_2_) was synthesized according to our previous study.^58^

### Characterization

An Agilent nuclear magnetic resonance (NMR) System VNMRS 500 Spectrometer was used for the ^1^H NMR analysis at room temperature in deuterated solvents with Si(CH_3_)_4_ as an internal standard. UV–vis analyses were performed on a Peak Instruments C-7000UV spectrophotometer with 1-cm path length cuvette, respectively. The molecular masses of the polymers were determined by two distinct gel permeation chromatography (GPC) systems using tetrahydrofuran (THF) and *N,N*-dimethyl formamide (DMF) as the eluent. In the first one, THF was utilized as the eluent at a flow rate of 1.0 mL min^–1^ at 40 °C on a Tosoh EcoSEC GPC system equipped with an autosampler system, a temperature controlled pump, a column oven, a refractive index (RI) detector, a purge and degasser unit, TSK gel superhZ2000, and a 4.6 mm ID × 15 cm × 2 cm column. The RI detector was calibrated with polystyrene and poly(methyl methacrylate) standards and GPC data were analyzed using EcoSEC Analysis software. A Tosoh EcoSEC dual detection (RI and UV) GPC system coupled to an external Wyatt Technologies Dawn Heleos-II multiangle light scattering detector and a Wyatt Technologies DynaPro NanoStar DLS detector was also used for size exclusion chromatography (SEC) measurements. DMF was used as the eluent at a flow rate of 0.5 mL/min at 45 °C. The column set was one Tosoh TSKgel G5000HHR column (7.8 × 300 mm), one Tosoh TSKgel G3000HHR column (7.8 × 300 mm), one Tosoh TSKgel SuperH-RC reference column for EcoSEC, and one Tosoh TSKgel HHR-H guard column (6 × 40 mm). Absolute molecular weights and molecular weight distributions were calculated using the Astra 7.1.2 software package.

### Synthesis of PBAE Diacrylate

Bis-acrylate functional PBAE was synthesized by aza-Michael addition-based poly-condensation polymerization.^22^ In brief, BDA (1.64 mL, 8.68 mmol) was taken into an opaque vial and AP (0.89 g, 8.68 mmol) was added. The reaction mixture was placed in a preheated oil bath at 100 °C with stirring. After 24 h, excess BDA (0.33 mL, 1.74 mmol) was added into the vial to obtain acrylate end-capped PBAE. After 3 h of further stirring at 100 °C, the reaction was cooled down to room temperature. The obtained polymer was dissolved in dichloromethane and precipitated in cold diethyl ether twice for the removal of residual monomers and oligomers. Then, the PBAE diacrylate was dried for 24 h at 40 °C under vacuum and stored at −20 °C until use. (*M*_w,GPC_(DMF): 7300 g/mol; *M*_w_/*M*_n,GPC_: 2.08; *M*_w,NMR_: 1830 g/mol; yield: 50%).

^1^H NMR (500 MHz, CDCl_3_, δ): 1.29–1.38 (br, m, NCH_2_CH_2_C*H*_2_CH_2_CH_2_OH), 1.43–1.48 (br, m, NCH_2_C*H*_2_CH_2_CH_2_CH_2_OH), 1.53–1.58 (br, m, NCH_2_CH_2_CH_2_C*H*_2_CH_2_OH), 1.68–1.77 (br, NCH_2_CH_2_(COO)CH_2_C*H*_2_), 2.38–2.52 (br, N(C*H*_2_)_3_), 2.73–2.84 (br, NCH_2_C*H*_2_(COO)CH_2_CH_2_), 3.58–3.66 (br, NCH_2_CH_2_CH_2_CH_2_C*H*_2_OH), 4.06–4.12 (br, NCH_2_CH_2_(COO)C*H*_2_CH_2_), 5.84 (2H, d, polymer-CH_a_ = CH_b_*H_c_*), 6.11 (2H, dd, polymer-CH_a_ = C*H_b_*H_c_), 6.40 (2H, d, polymer-C*H*_a_ = CH_b_H_c_).

### End-group Transformation of PBAE Diacrylate to Norbornene

PBAE diacrylate (1.21 g, *M*_w,GPC_: 7300 g/mol, 0.166 mmol) was dissolved in THF (5 mL). After the addition of 5-nonbornene-2-methyl amine (NB-NH_2_) (215 μL, 1.68 mmol), the reaction solution was stirred at room temperature for 24 h. The modified polymer was precipitated in diethyl ether twice. After being dried under vacuum for 24 h, the norbornene functional PBAE (NB-PBAE-NB) was obtained. (Transformation: >98%, confirmed by NMR).

^1^H NMR (500 MHz, CDCl_3_, δ): 1.29–1.38 (br, m, NCH_2_CH_2_C*H*_2_CH_2_CH_2_OH), 1.43–1.48 (br, m, NCH_2_C*H*_2_CH_2_CH_2_CH_2_OH), 1.53–1.59 (br, m, NCH_2_CH_2_CH_2_C*H*_2_CH_2_OH), 1.68–1.77 (br, NCH_2_CH_2_(COO)CH_2_C*H*_2_), 2.37–2.46 (br, N(C*H*_2_)_3_), 2.73–2.79 (br, NCH_2_C*H*_2_(COO)CH_2_CH_2_), 2.85 (4H, br, N(C*H*_2_)-norbornene), 3.58–3.64 (br, NCH_2_CH_2_CH_2_CH_2_C*H*_2_OH), 4.06–4.14 (br, NCH_2_CH_2_(COO)C*H*_2_CH_2_), 5.96–6.25 (4H, d, −C*H*=C*H*– (norbornene)).

### Synthesis of 2-Deoxy-2-methacrylamido-d-glucose

2-Deoxy-2-methacrylamido-d-glucose (MAG) was synthesized according to a published procedure.^59,60^ Briefly, D-(+)-glucosamine hydrochloride (10.0 g, 46 mmol) was dissolved in 250 mL of methanol containing potassium carbonate 6.41 g (46 mmol) in a 500-mL single-neck round-bottom flask with vigorous stirring, then the mixture was cooled down to −10 °C with an acetone/ice bath. Afterward, methacryloyl chloride (4.0 mL, 41 mmol) was added drop wise into the mixture, and the mixture was stirred at −10 °C for 30 min. After another stirring for 3 h at room temperature, the precipitated white salt was filtered off from the crude product using a sintered funnel with vacuum suction. A white slurry was obtained after concentration of the filtrate on a rotary evaporator. The product was purified by a column chromatography using dichloromethane/methanol (4:1) as the eluent. (Yield: 37%).

^1^H NMR (500 MHz, D_2_O, δ): 2.00 (3H, s, CH_2_=C(C*H*_3_)), 5.54 (1H, sd, CH*H*=C(CH_3_)), 5.76 (1H, sd, CH*H*=C(CH_3_)), 3.50–3.58 (m, 5-H_β_, 4-H_αβ_), 3.65–3.71 (m, 3-H_β_), 3.78–3.98 (m, 2-H_β_, 3-H_α_, 6-H_αβ_), 4.00 (5-H_α_), 4.02 (dd, 2-H_α_), 4.84 (d, 1-H_β_), 5.29 (d, 1-H_α_).

### Synthesis of P(MAG-*co*-HEMA) by RAFT Polymerization

Poly(2-deoxy-2-methacrylamido-d-glucose-*co*-2-hydroxyethyl methacrylate) [P(MAG-*co*-HEMA)] was synthesized via RAFT polymerization^61^ with a molar ratio of reagents [MAG]:[HEMA]:[CTA]:[AIBN] = 12:12:1:0.25. MAG (923 mg, 3.73 mmol), 2-HEMA (486 mg, 3.73 mmol), 2,2′-AIBN (12.8 mg, 0.079 mmol), and 4-cyano-4-((dodecyl-sulfanylthiocarbonyl)sulfanyl) pentanoic acid (CTA) (126 mg, 0.31 mmol) were dissolved with DMF, (9 mL), respectively, in a Schlenk tube equipped with a magnetic stir bar. The polymerization solution was degassed via three freeze-pump-thaw cycles, refilled with nitrogen, and then stirred in an oil bath at 70 °C for about 18 h. After 18 h, the flask was cooled and the solution was poured into a 20 times excess of THF. The precipitate was filtered off and dried under vacuum. The monomer conversion was gravimetrically determined as 88%. To remove unreacted monomer and other impurities, the polymer was dialyzed against distilled water using dialysis membrane with a molecular weight cutoff (MWCO) of 3500 Da. Then, the solution was lyophilized to yield P(MAG-*co*-HEMA) as white powder. (*M*_n,GPC_: 10,950 g/mol; *M*_w_/*M*_n,GPC_: 1,02; *M*_n,theo_: 3900 g/mol; yield: 88%).

### End-Group Transformation of P(MAG-*co*-HEMA) to Tetrazine

Carboxylic acid end of P(MAG-*co*-HEMA) was activated by EDC and NHS, and reacted with amino tetrazine (Tz-NH_2_). Briefly, P(MAG-*co*-HEMA) (1.0 g, 9.1 × 10^–5^ mol) was dissolved in 10 mL of dimethyl sulfoxide (DMSO) and the carboxylic acid group was activated using EDC (98 mg, 5.1 × 10^–4^ mol) in the presence of NHS (35 mg, 3.0 × 10^–4^ mol) and triethyl amine (Et_3_N) (43 μL, 3.1 × 10^–4^ mol). The reaction mixture was stirred at 25 °C for 24 h. Afterward, Tz-NH_2_ (155 mg, 7.7 × 10^–4^ mol) dissolved in DMSO was added dropwise to the reaction mixture. After 24 h of stirring, the reaction mixture was precipitated and washed twice with THF. Then, the pink polymer (P(MAG-*co*-HEMA)-Tz) was dried overnight at 40 °C under vacuum and stored at −20 °C until use.

### Synthesis of PMAG-*co*-2-HEMA)-*b*-PBAE) [P(MAG-*co*-HEMA)-*b*-PBAE]

The block copolymer was prepared via the IEDDA click reaction. The norbornene functional PBAE (NB-PBAE-NB) (445 mg, 5.9 × 10^–5^ mol) was dissolved in 3 mL of DMSO in a vial equipped with a magnetic stirrer. The tetrazine functional P(MAG-*co*-HEMA)-Tz (618 mg, 5.6 × 10^–5^ mol) was added into this solution in two portions (75% + 25% by mass) to follow the reaction with UV–vis spectroscopy. At specific time intervals, the UV–vis spectra were recorded. After the completion of reaction (about 37 h), the reaction mixture was precipitated and washed twice with diethyl ether containing a small amount of ethanol. After being dried under vacuum for 24 h, the block copolymer [P(MAG-*co*-HEMA)-*b*-PBAE] was received. (Recovery: 63%).

### pH-Sensitive Behavior of the Polymers

pH sensitivity of the norbornene functional PBAE and the block copolymer [P(MAG-*co*-HEMA)-*b*-PBAE] was evaluated by acid–base potentiometric titration and measurement of optical density (OD) of the solutions.^62^ For this, 6.4 mg of the copolymer was dispersed in 3 mL distilled water and the pH was adjusted to 3 by the addition of small aliquots of 0.1 M HCl. Then, the polymer solution was titrated by the addition of 0.1 M NaOH, and at each step, the pH and OD (at 550 nm) of the solution were measured by a pH-meter and UV–vis spectrophotometer, respectively. To determine the base dissociation constant (p*K*_b_), OD values and volumes of NaOH solutions were plotted against pH values.^37^

### Preparation of Blank Micelles

The micelles were obtained via consecutive acid and base addition to an aqueous dispersion of the block copolymer. Briefly, P(MAG-*co*-HEMA)-*b*-PBAE (8.3 mg) dissolved in minimal amount of DMSO was dispersed in 2.5 mL of distilled water, then 0.1 HCl was added under stirring to adjust pH 3. After the addition of the acid, the turbid mixture became clear, so that the polymer was dissolved completely. Then, 0.1 M NaOH solution (∼0.5 mL) was slowly added in a dropwise manner under stirring till the pH was around 9. The solution became cloudy indicating the formation of micelles. The mixture was dialyzed against distilled water using a dialysis membrane with an MWCO of 3500 Da. In the end, the micelles were obtained.

### Preparation of DOX-Loaded Micelles

DOX, which was chosen a model drug, was encapsulated into the micelles using a similar method. A typical procedure for drug loading is as follows. First, 70 mg of the block copolymer and 7 mg of DOX hydrochloride dissolved in a minimal amount of DMSO (∼600 μL) was added to 2.5 mL distilled water, and pH was adjusted to 3 by the addition of 0.1 M HCl. Then 0.1 M NaOH slowly added dropwise under stirring and pH was adjusted to 9 by the addition of 0.1 M NaOH. The solution was dialyzed against distilled water using a membrane (MWCO 3500 Da) for a day at room temperature. The water was replaced with fresh water six times. DOX-loaded red solid polymeric micelles were obtained after lyophilization.

The drug loading capacity (DLC) and drug loading efficiency (DLE) of the polymeric micelles were determined using eqs 1 and 2, respectively.^63^ In a representative example, 2.8 mg DOX-loaded micelles were solved in DMSO (8 mL); thus, the micelles were broken and the encapsulated DOX came out and was solubilized. Then, the absorbance at 483 nm was recorded by UV–vis spectroscopy. The DOX content of the micelle was determined by using a calibration curve established with absorbance values of DOX solutions of various concentrations at the same wavelength (483 nm).
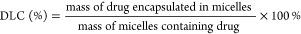
1
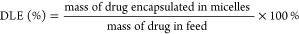
2

### Characterization of Micelles

Micelles (1 mg/mL) were dropped on a carbon film-coated Cu grid and left to dry overnight. Samples were imaged on a Thermo Scientific Quattro ESEM scanning electron microscope using a scanning transmission electron microscopy (STEM) detector under a high vacuum (30 kV) from a working distance of 7.7 mm, and the digital images of micelles were captured to analyze their morphology. Dynamic light scattering (DLS) (Malvern Zetasizer Nano ZS, Malvern Instruments, UK) was performed to determine the average size and size distribution, and electrophoretic light scattering was performed to determine the zeta potential of the prepared micelles. The critical micelle concentration (CMC) of the block copolymer with a variety of concentrations ranging from 10 mg/mL to 3 × 10^–6^ mg/mL was measured by DLS. All DLS measurements were carried out at 25 °C and repeated three times. The CMC of the polymer was estimated by plotting count rate (kcps) as a function of concentration. The intersection of the upper and lower linear trend lines imply the CMC.^64^

### In Vitro Release of DOX from Polymeric Micelles

The release profiles of DOX from polymer micelles were studied using a dialysis method in 0.01 M phosphate buffered saline (PBS) at pH 7.4 and 5.3. In a typical drug release study, a solution (1 mL) of DOX-loaded polymeric micelles (1.5 or 1.9 mg/mL) in PBS (pH 7.4, 0.01 M) was dialyzed in a dialysis membrane (MWCO 3500 Da) against 30 mL of PBS (pH 7.4, 0.01 M or pH 5.3, 0.01 M) containing Tween 80 (1% or 0.33% w/v). At specific time intervals, 1 mL of buffer solution outside the dialysis membrane was withdrawn and replaced with an equal volume of fresh PBS buffer. The amount of DOX released from the micelles was determined by measuring absorbance at 483 nm using a UV–vis spectrophotometer. The cumulative release of DOX was calculated by using the following equation:^65^
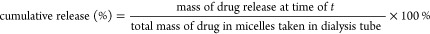
3

### Cell Viability Assay

The HUVEC cell line and glioblastoma cell line U87-MG were used to evaluate the drug release performance of polymeric micelles by using MTT (3-(4,5-dimethylthiazol-2-yl)-2,5-diphenyltetrazolium bromide) assay. Micelles with DOX (EK255), without DOX (EK257), and only DOX groups were tested in triplicate. Drug concentrations ranging from 20 to 0.625 μg/mL were tested for 12, 24, and 48 h of treatment.

The protocol was summarized as follows: 10,000 cells were seeded into a sterile 96-well plate and incubated at 37 °C for 24 h in an incubator with 5% CO_2_ and 95% humidity. Later, the medium was removed and the micelle containing cell media was added and incubated for 12, 24, and 48 h. Later, 10 μL of 5 mg/mL MTT solution was added to each well and incubated for 3 h at 37 °C. Finally, 100 μL of solubilization buffer was added to each well to dissolve the formazan crystals formed and additional 15 min of incubation was done at room temperature. After incubation, absorbance was measured at a wavelength of 570 nm in a Hidex Sense microplate reader. Percent cell viability scores were evaluated by normalizing the data to untreated cells on the corresponding day of incubation.

### Cellular Uptake Assay

U87-MG cells were seeded on a 6-well plate (25 × 10^4^ cells/well) and incubated at 37 °C with 5% CO_2_ for 24 h. Cells were subjected to DOX (5 μg/mL), DOX-loaded micelles (EK255) (final DOX concentration is 5 μg/mL), and free micelle groups (EK257) for 4 h. The cells were washed three times with 0.1% PBS-T. The cells were stained by 4′,6-diamidino-2-phenylindole (DAPI) for 2 min and washed three times with 0.1% PBS-T. Fluorescence imaging (Leica DM2500) was used to visualize and five different photographs of each group were taken. Excitation wavelengths were 320–380 nm for DAPI and 515–560 nm for DOX. Cellular uptake was analyzed by counting the DAPI and DOX containing cells on photographs. Percent cellular uptake was calculated as the ratio of all counted cells to cells with double positive staining (DAPI and DOX positive).

### Annexin V Staining

APC Annexin V Apoptosis Detection Kit with propidium iodide (PI) (Biolegend, San Diego, USA) was used to determine cell death. Briefly, U87-MG cells were seeded on a 6-well plate at a density of 25 × 10^4^ cells per well and incubated at 37 °C with 5% CO_2_ overnight. The cells were treated with 5 μg/mL of DOX, DOX-loaded micelle (EK255), and empty micelle (EK257) groups for 2 and 4 h. Cells were harvested and the pellets were re-suspended in 100 μL of 1× Annexin V binding buffer. The cells were then incubated with 5 μL of Annexin V-FITC and 10 μL of PI for 15 min in the dark at room temperature and 400 μL of 1× Annexin V binding buffer was added, according to the manufacturer’s instructions. Cell fluorescence was measured by flow cytometry (NovoCyte, ACEA Biosciences Inc., CA, USA). The ratio of cell death was assessed with single PI positive cells (Q2–1). Early apoptosis and late apoptosis were detected by single APC positive cells (Q2–4) and APC and PI double positive (Q2–3) cells, respectively.

## Results and Discussion

### Synthesis and Characterization of the Polymers

**Scheme 1:**
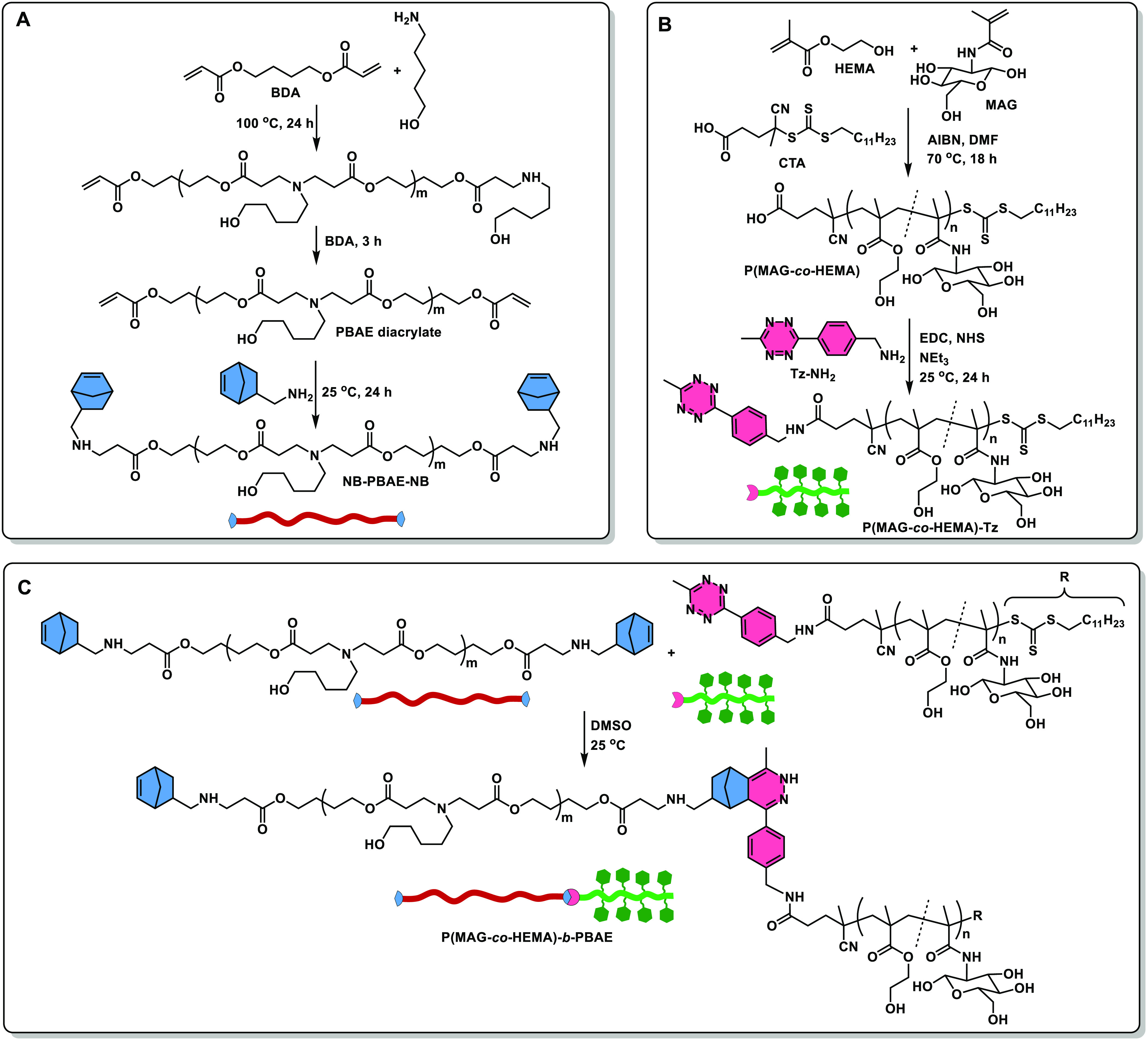
Synthetic Approach for the Preparation of the NB-PBAE-NB (A), P(MAG-*co*-HEMA) (B), and P(MAG-*co*-HEMA)-*b*-PBAE (C)

P(MAG-*co*-HEMA)-*b*-PBAE was synthesized as shown in Scheme 1 for the construction of pH-responsive, biodegradable micelles containing glucose moieties that potentially target specifically cancer cells. The IEDDA click reaction was chosen for the conjugation of the glycopolymer and the PBAE due to its kinetics and orthogonality. IEDDA has been intensively and effectively employed in live-cell imaging,^66−68^ diagnostics,^69,70^ chemical biology,^71,72^ biomaterials,^73−75^ material science,^76,77^ and polymer science.^58,78−80^ First, the pH-responsive hydrophobic PBAE segment was synthesized via Michael addition polymerization of relatively hydrophobic monomers, namely, BDA and AP. At the final stage of the polymerization, the addition of excess BDA yielded acrylate end-capped PBAE, then, it was converted to norbornene end functional PBAE via reacting the terminal acrylates with amino norbornene (Scheme 1-A). The molar mass of the PBAE diacrylate was determined by GPC and NMR as *M*_w,GPC_: 7300 g/mol and *M*_n,NMR_: 1830 g/mol. End-group transformation from acrylate to norbornene was confirmed by ^1^H NMR spectroscopy. As seen from the NMR spectrum of PBAE diacrylate (Figure S1), the signals around 4.10 to 3.60 ppm were characteristic of protons of methylene groups neighboring oxygen atoms. The peaks belonging to protons of methylene groups adjacent to nitrogen and carbonyls appeared at 2.45 and 2.80 ppm, respectively. The peaks between 1.29 and 1.77 ppm were attributed to the aliphatic protons of the side chains. The most specific peaks observed at 5.84, 6.11, and 6.40 ppm were ascribed to terminal acrylate protons. Those peaks due to the acrylate functionality disappeared after the Michael addition reaction of amino norbornene with the acrylates, while new peaks of olefin protons of norbornene moieties appeared at 5.96–6.25 ppm with their distinctive shape (Figure S2). The structures of the PBAEs were further confirmed by Fourier transform infrared (FTIR) spectra as shown in Figure S3. The broad bands centered at 3434 cm^–1^ were attributed to the stretching of O–H groups, and C–H stretching bands were observed at 2930 and 2860 cm^–1^. Strong bands at 1725 and 1170 cm^–1^ belonging to C=O and C–O, respectively, supported the PBAE structure. Furthermore, the small band at 1638 cm^–1^ was considered to be due to C=C stretching vibrations of terminal acrylate and norbornene groups.

**Figure 1:**
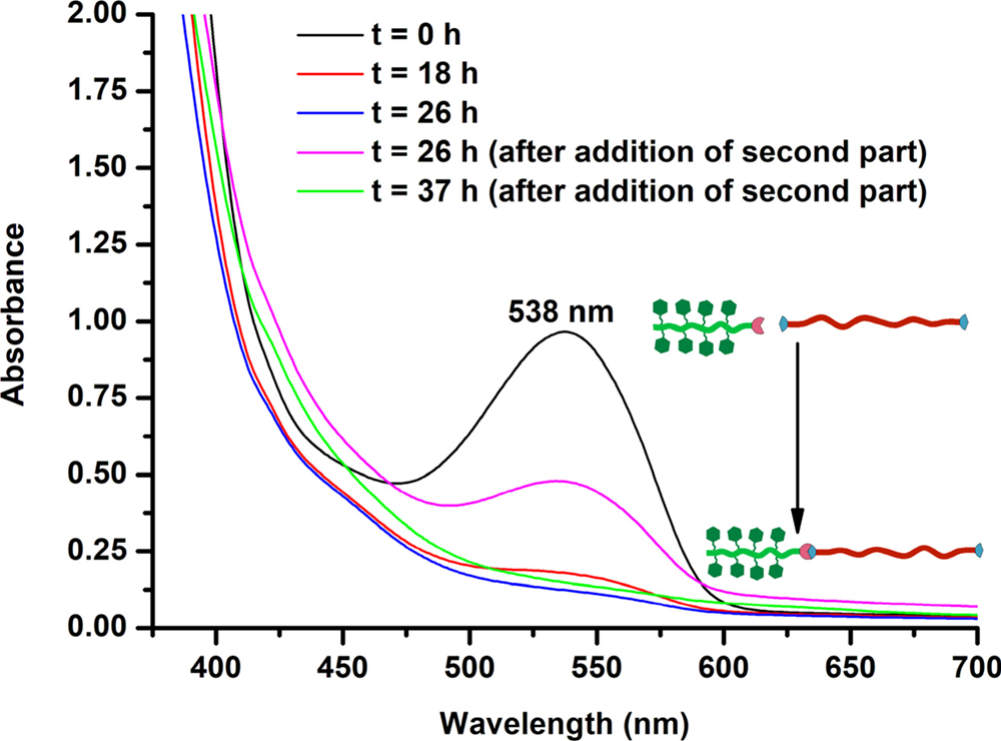
UV–vis spectra of the solution containing NB-PBAE-NB and P(MAG-*co*-HEMA)-Tz in DMSO at specific time intervals during the formation of P(MAG-*co*-HEMA)-*b*-PBAE via the tetrazine mediated IEDDA click reaction. P(MAG-*co*-HEMA)-Tz was added in two portions at *t* = 0 h and *t* = 26 h.

**Figure 2:**
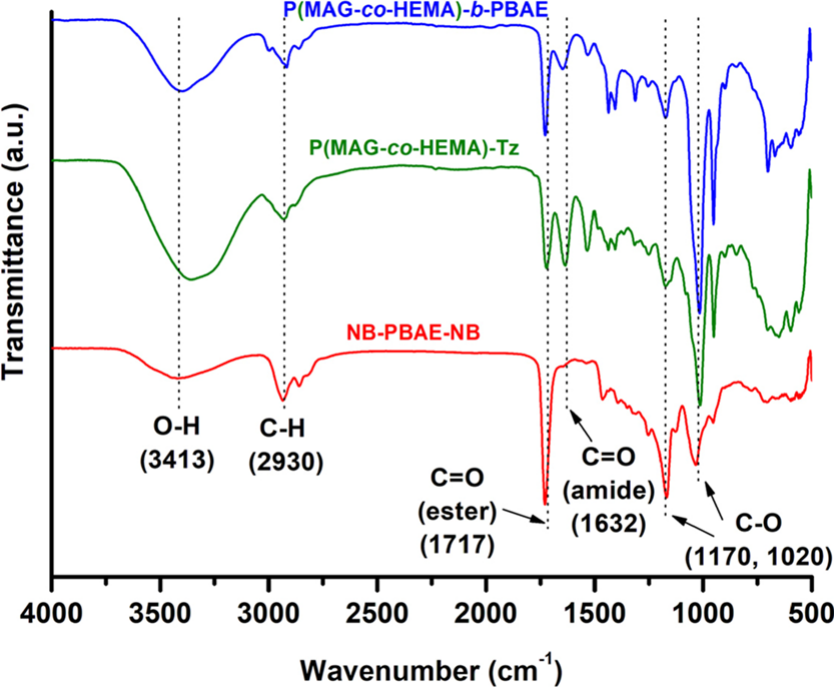
FTIR spectra of NB-PBAE-NB, P(MAG-*co*-HEMA)-Tz and P(MAG-*co*-HEMA)-*b*-PBAE.

**Figure 3:**
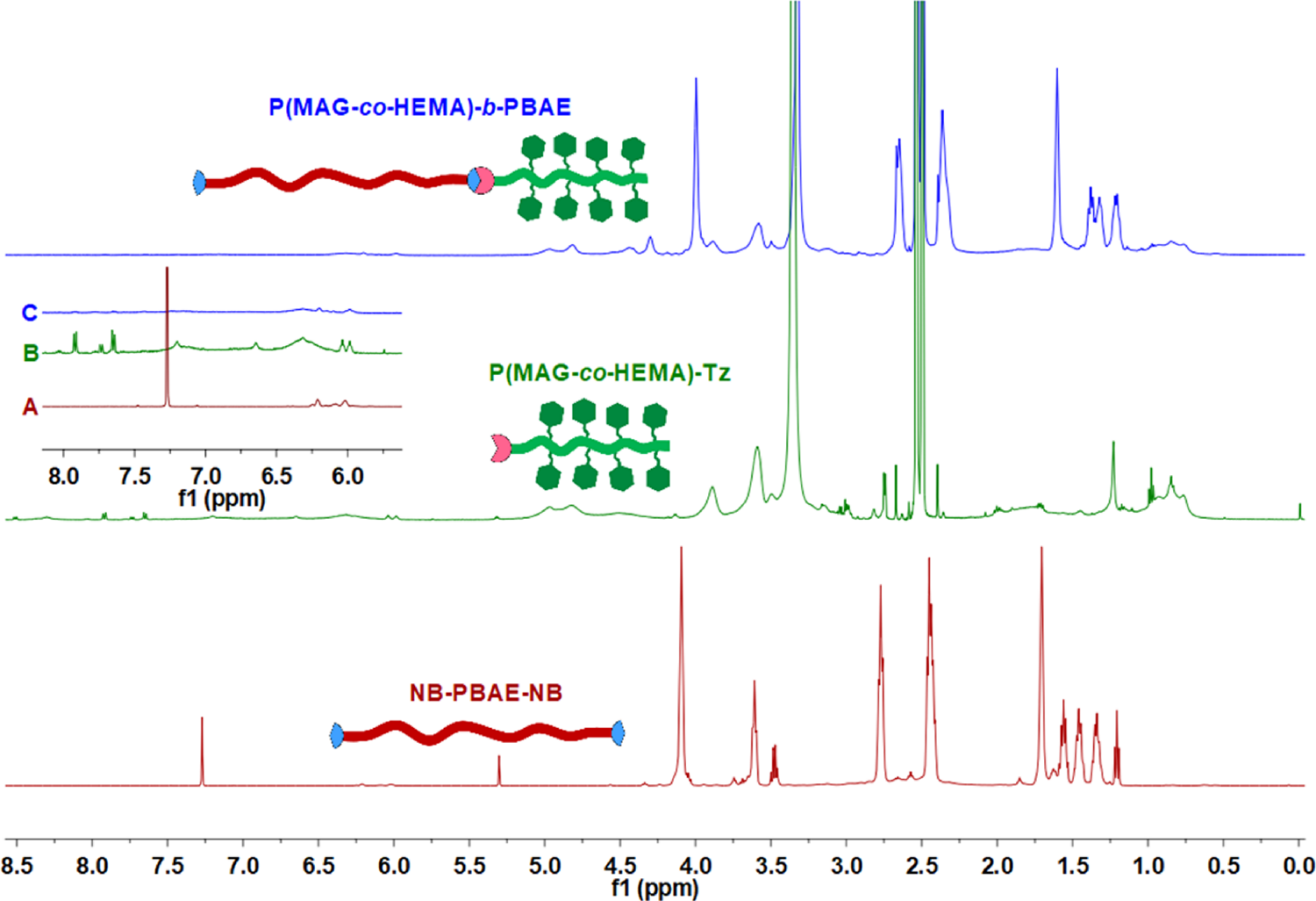
^1^H NMR spectra of NB-PBAE-NB (A), P(MAG-*co*-HEMA)-Tz (B), and P(MAG-*co*-HEMA)-*b*-PBAE (C) (see Figures S2, S6 and S8 in the Supporting Information for peak assignments).

P(MAG-*co*-HEMA) was chosen as the hydrophilic segment bearing glucose groups. The copolymer was synthesized via the RAFT polymerization of 2-HEMA and MAG with a molar ratio of [MAG]:[HEMA]:[CTA]:[AIBN] = 12:12:1:0.25 (Scheme 1B). The molecular mass and polydispersity index were determined by aqueous GPC as *M*_n,GPC_: 10,950 g/mol and *M*_w_/*M*_n,GPC_: 1,02, respectively. The molecular mass determined by GPC was considerably different than the theoretical value (3900 g/mol). A similar behavior, the higher molecular masses by GPC than theoretical values, was observed by the others.^61^ This difference can be related to conformational states of glycopolymer coils^61^ or the lower chain transfer coefficient^60^ in the polymerization. The chemical structure of P(MAG-*co*-HEMA) was analyzed with ^1^H NMR spectroscopy (Figure S5). The most typical proton signals of comonomers, MAG and HEMA, were observed at 5.04 and 3.99 ppm, respectively, which were attributed to the anomeric proton signals of the sugar molecules^61^ and (−O–***CH*_*2*_**–CH_2_–OH) signals of HEMA moieties. After the conjugation of the polymer with amino tetrazine (Tz-NH_2_), aromatic proton signals of the tetrazine groups appeared at 7.66 and 7.92 pm. Furthermore, the appearance of new bands (1438, 1406, and 952 cm^–1^) was attributed to tetrazine moieties^81^ in the FTIR spectrum; the typical pink color of the polymer and an absorbance band centered at 538 nm in the UV–vis spectrum of the polymer (Figure 1, spectrum at *t* = 0 h) supported the incorporation of the tetrazine functionalities on the polymer. The tetrazine functional polymer was then utilized in the fabrication of P(MAG-*co*-HEMA)-*b*-PBAE via the tetrazine mediated IEDDA click reaction (Scheme 1C). The reaction was performed by the addition of P(MAG-*co*-HEMA)-Tz (in two portions 75% + 25% by mass) into a solution of NB-PBAE-NB to assure the formation of the AB block copolymer and to follow the reaction with UV–vis spectroscopy. The click reaction was readily followed by tracking the disappearance of absorbance at 538 nm by the tetrazine group (Figure 1). After complete disappearance of the absorbance band, P(MAG-*co*-HEMA)-*b*-PBAE was obtained. The structure of the block copolymer was confirmed by FTIR and NMR spectroscopy. The stretching band of O–H, C–H, and C–O was observed at 3413, 2930, and 1170 and 1020 cm^–1^, respectively, in all spectra (Figure 2). Specifically, a sharp stretching band of ester carbonyls of NB-PBAE-NB appeared at 1717 cm^–1^; while, the spectrum of P(MAG-*co*-HEMA) contained two carbonyl bands at 1717 and 1632 cm^–1^ ascribed to the ester and the amide linkages, correspondingly. As compared to that of the precursors, the ester carbonyl band (1717 cm^–1^) became stronger in the spectrum of the block copolymer, P(MAG-*co*-HEMA)-*b*-PBAE. In addition, the appearance of the characteristic peaks of both segments, shift of the aromatic proton peaks of the tetrazine (7.66 and 7.92 pm), and norbornene peaks (5.96–6.25 ppm) implied the block copolymer formation (Figures 3 and S8).

For further insight, molecular masses of the polymers were analyzed with aqueous GPC. As shown in Figure S9, P(MAG-*co*-HEMA) had almost a unimodal GPC trace corresponding to *M*_n_ = 10,950 g/mol with a low polydispersity index (*M*_w_/*M*_n_ = 1.02). After the click reaction, the maxima in both the RI signal and light scattering signal were shifted to higher molecular mass of 36,180 g/mol (*M*_w_/*M*_n_ = 1.44). The increase in molecular mass was higher than the expected one probably due to the incorporation of a segment with a completely different nature. This result supported the formation of the block copolymer.

### pH-Sensitive Behavior of the Polymers

**Figure 4:**
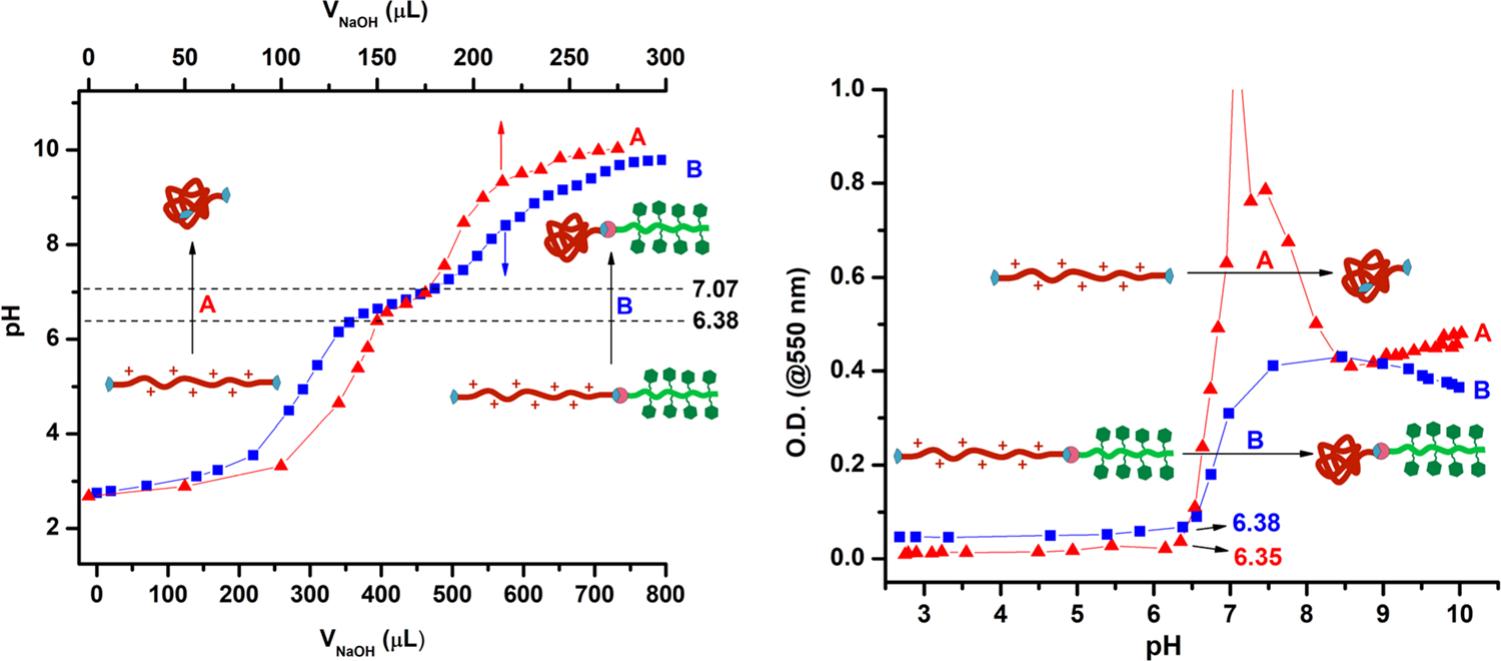
Titration curves (left) and pH-dependent absorbance (right) of NB-PBAE-NB (A) and P(MAG-*co*-HEMA)-*b*-PBAE (B).

To confirm the pH sensitivity of the PBAE and the block copolymer [P(MAG-*co*-HEMA)-*b*-PBAE], acid–base titration was performed with simultaneous pH and OD measurements.^62^ Before titration, both polymers were dispersed in distilled water resulting in a turbid mixture. When the pH values of the mixtures were adjusted to be around 3 by addition of the acid, the solutions became clear due to the hydrophobic/hydrophilic transition in the PBAE segment. The amine groups on the polymers were protonated; thus, the hydrophobic PBAE became hydrophilic and soluble in aqueous solution. These clear solutions were titrated by the addition of small aliquots of NaOH solution (0.1 M). Figure 4 shows both the titration curves (left) and the change in OD against pH during the titration. First, pH changed rapidly with the addition of NaOH (pH 3–6) in the titration curves (Figure 4, left). Then, change in pH slowed down in the range 6.38–7.07 in which tertiary amine groups of PBAE chain were deprotonated gradually. The p*K*_b_ values of the NB-PBAE-NB and P(MAG-*co*-HEMA)-*b*-PBAE were calculated by the determination of inflection point in the derivative of the titration curves as 6.74 and 6.75, respectively.^37,62,82^ The OD curves (Figure 4, right) supported the protonated/deprotonated transition of the PBAE segments. At acidic pH below p*K*_b_, ODs were low since the PBAE segment was protonated and fully soluble; while, the PBAE segments were deprotonated above the p*K*_b_ and became insoluble leading to higher turbidity. As a result, P(MAG-*co*-HEMA)-*b*-PBAE may form a stable micelle at a physiological pH of 7.4 and exhibit a pH-sensitive hydrophobic/hydrophilic transition in the tumor microenvironment around pH 5.5.^37^ The micelles obtained from P(MAG-*co*-HEMA)-*b*-PBAE above pH of 7 can be broken gradually around pH 5.5 and release the encapsulated hydrophobic drugs (i.e., DOX). Therefore, such pH-sensitive block copolymers with the PBAE segment are good candidates for anticancer drug carriers.^83,84^

### Preparation and Characterization of the Micelles

**Figure 5:**
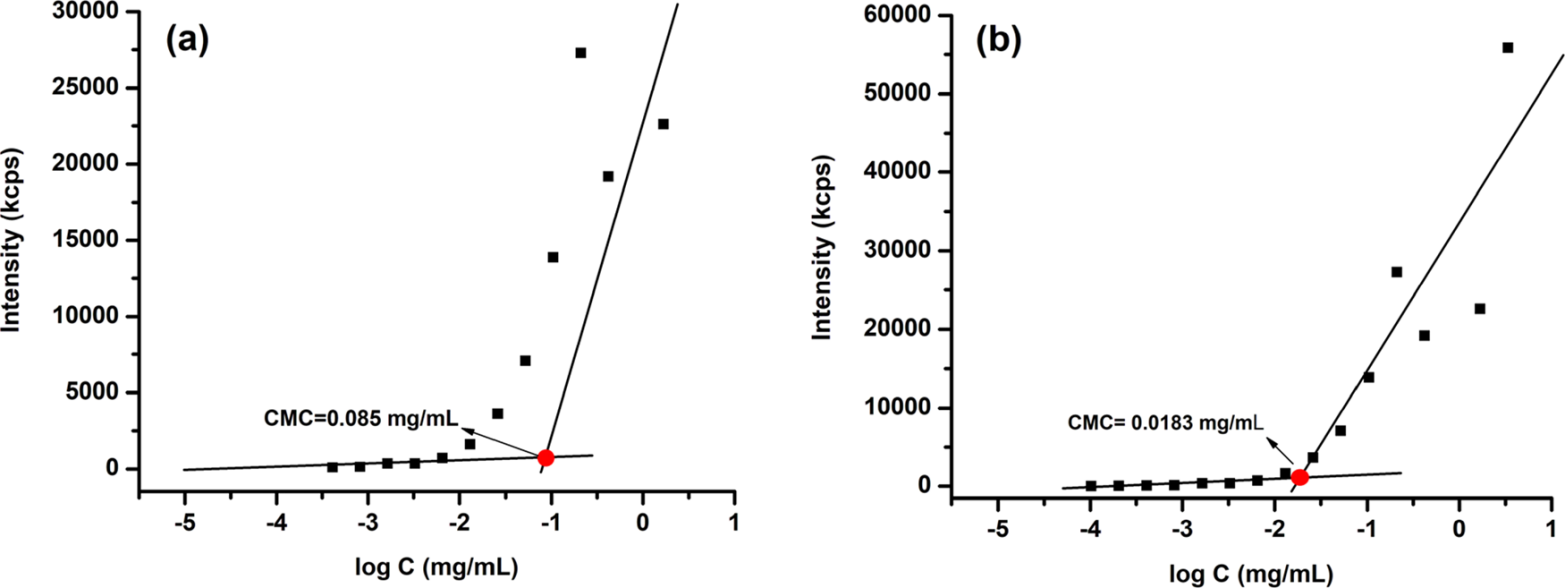
CMC estimation for P(MAG-*co*-HEMA)-*b*-PBAE with (a) or without (b) DOX by plotting the count rate (kcps) as a function of concentration on a DLS device.

The pH-sensitive amphiphilic block copolymer P(MAG-*co*-HEMA)-*b*-PBAE was utilized to form core–shell self-assembled micelles as the DOX carrier with cancer cell targeting potential. First, the CMC of the amphiphilic polymer with or without DOX was estimated by plotting count rate (kcps) as a function of concentration on a DLS device. The scattering intensities detected for P(MAG-*co*-HEMA)-*b*-PBAE concentrations below CMC were constant corresponding to that of deionized water. The CMC of the blank and drug-loaded micelles was estimated as 0.085 mg/mL and 0.0183 mg/mL, respectively, from the intersections of the upper and lower linear trend lines in the plots (Figure 5).

**Figure 6:**
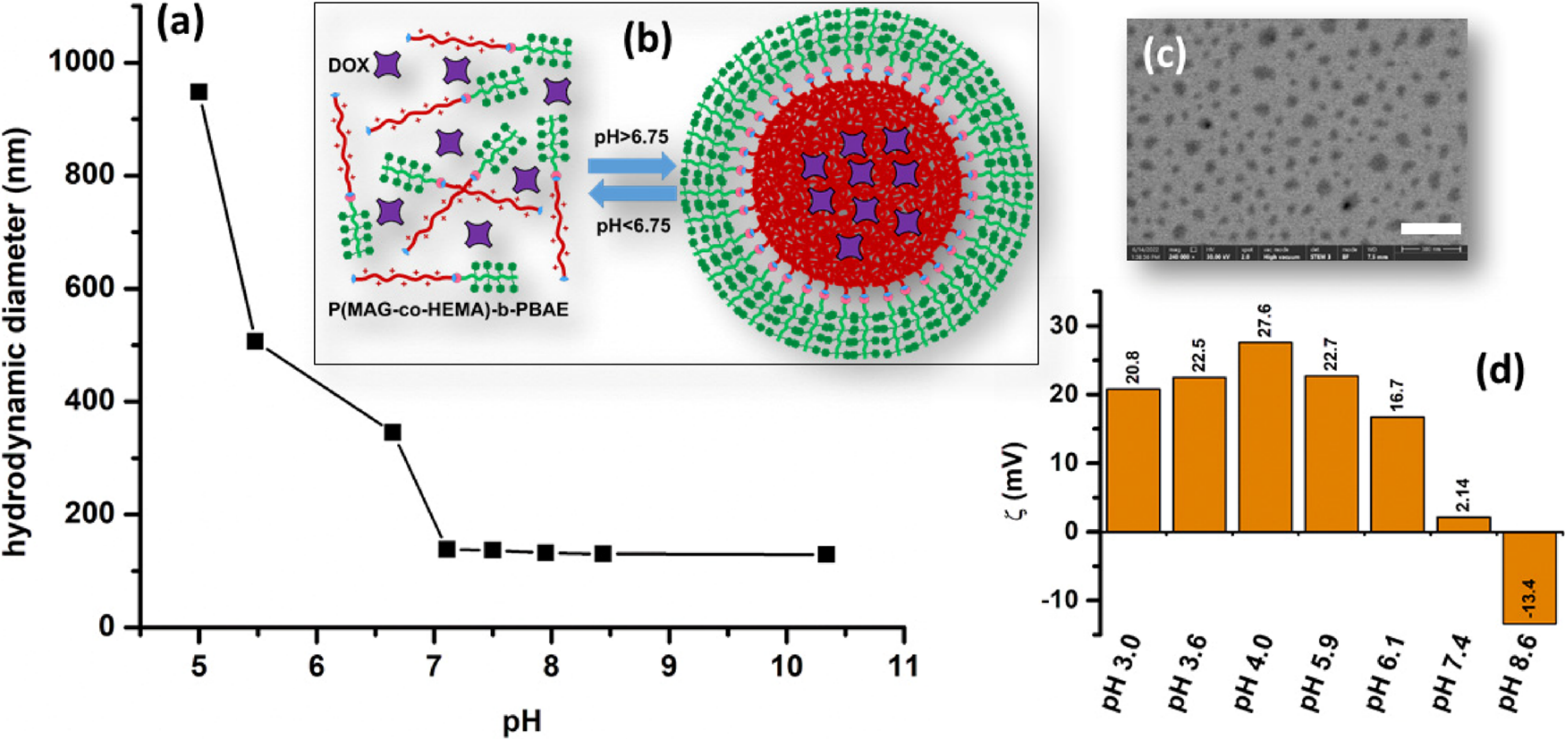
(a) Change of the hydrodynamic diameter of blank micelles with pH; (b) schematic representation of the micelle formation via self-assembly above p*K*_b_ (6.75); (c) STEM image of the DOX-loaded micelles (scale bar: 300 nm); (d) zeta potentials (ζ) of the polymer at various pH.

Then, both the blank and the DOX-loaded micelles were prepared by a modified dialysis method.^85^ Briefly, the block copolymer or the block copolymer/DOX hydrochloride was dissolved in a minimal amount of DMSO (∼300 μL) and dispersed in distilled water. After adjusting the pH to 3, the mixture turned into a clear homogeneous solution, since the PBAE segment was protonated and whole polymer became soluble at low pH values (below p*K*_b_). Subsequently, the slow addition of dilute NaOH solution induced the protonated/deprotonated or hydrophilic/hydrophobic transition of PBAE at pH higher than p*K*_b_ (6.75) resulting in self-assembly of the polymers into micellar structures (Figure 6b) with a diameter of 179 nm (blank) and 174 nm (DOX-loaded) (Figure 6a). The STEM image (Figure 6c) supported the formation of drug-loaded micelles with a size of 60.3 nm (±8.4 nm) (dried). The reverse transition (deprotonated/protonated) was observed through hydrodynamic diameter (*d*_micelle_) measurements on DLS at different pH as shown in Figure 6a. The hydrodynamic diameter increased dramatically when pH decreased, since the micelles were swollen and then broken. Moreover, zeta potential (ζ) measurements above and below p*K*_b_ supported this transition as shown in Figure 6d. Around physiological pH (pH 7.4), the zeta potential of the polymeric micelles showed very low positive charge (+2.14 mV), and it was negative under basic conditions such as −13.4 mV at pH 8.5. In contrast, under acidic conditions, the zeta potentials were sharply increased taking the values of +20.8–+27.6 mV in the pH range of 3.0–5.9, because the amine residues of the PBAE segment were fully protonated yielding positively charged quaternary amine residues.^85^ The micelles showed a relatively low zeta potential of +16.7 mV at pH 6.1, which was close to p*K*_b_ (6.75), due to partial protonation of the tertiary amines.

The DOX content of the lyophilized micelle was determined by using a calibration curve established with absorbance values of DOX solutions of various concentrations at the same wavelength (483 nm). The amount of DOX encapsulated by 70 mg of micelles was determined as 6.2 mg; as a result, DLC (%) and DLE (%) were found to be 9 and 89%, respectively.

### In Vitro Release of DOX from Polymeric Micelles

**Figure 7:**
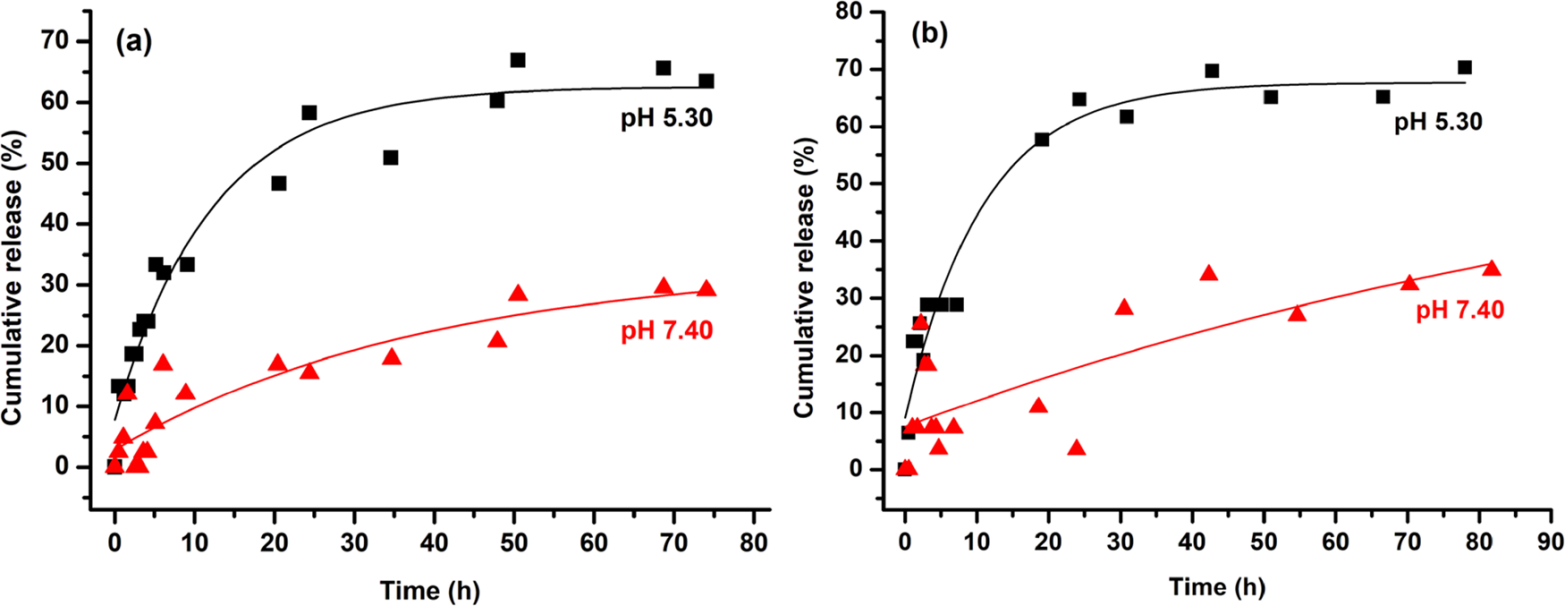
Release profiles of DOX from DOX-loaded micelles at different pH of 5.30 and 7.40 in the presence of Tween 80 (a: 1%; b: 0.33% by mass). Release profiles were measured by UV–vis spectrophotometry.

As polymeric micelles exhibited a pH-responsive property, the in vitro drug release performance of the micelles was tested at physiological (PBS, 0.01 M, pH 7.4) and acidic pH (PBS, 0.01 M, pH 5.30) as shown in Figure 7. It can be found obviously that the DOX release rates from the particles were significantly changed at different pH values. The micelles at pH 5.30 had a higher release rate and amount of DOX compared to those at pH 7.40. The improved release at lower pH was attributed to disassembly of the micelles due to the hydrophobic/hydrophilic transition of the PBAE segment.

### Cell Viability Assay

**Figure 8:**
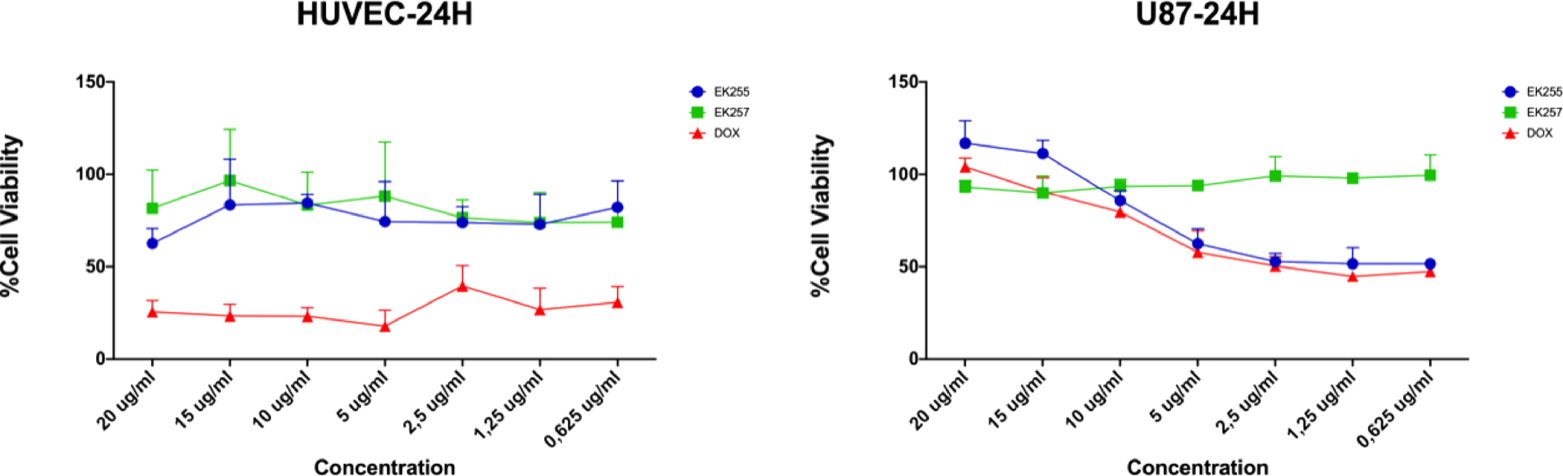
Cell viability assay with HUVEC and U87-MG cell lines for 24 h of treatment. EK255: DOX-loaded micelle; EK257: Micelle without DOX: Free DOX.

The optimum efficiency of the DOX-loaded micelles was obtained in 24 h of incubation. In noncancerous HUVEC cells, DOX treatment in all tested concentrations killed the cells whereas micelles with or without DOX were not toxic for cells. However, DOX-loaded micelles (EK255) significantly reduced cell viability whereas micelles without DOX (EK257) did not significantly affect U87-MG cell viability (Figure 8). The results indicated that DOX encapsulation specifically targeted the cancer cells and reduced the cell viability within 24 h of incubations] whereas noncancerous cells were not affected by the micelle treatment.

Polymeric micelle encapsulation increased the specific activity of DOX induced cytotoxicity. While polymeric micelles are not cytotoxic to both cancer and noncancer cells, when they are loaded with an anticancer drug, they specifically targeted cancer cells. Previous studies with similar approaches including micelle and DOX treatments also reported reduced cell viability on several cancer cells HeLa, HepG2^85^ and MCF-7^82^ cells. However, in our study, we reported that the toxic effect of DOX encapsulated into polymeric micelles was similar to only DOX treatment in U87-MG cells with better efficiency after 24-hour incubation.

In addition, it was observed that the release of DOX from micelles provided higher toxicity, especially at a concentration below 5 μg/mL. It implies that, besides changes in pH,^82,85^ the drug concentration in the micelle is also effective in releasing hydrophobic drugs (like DOX) in U87-MG cells.

### Cellular Uptake Assay

**Figure 9:**
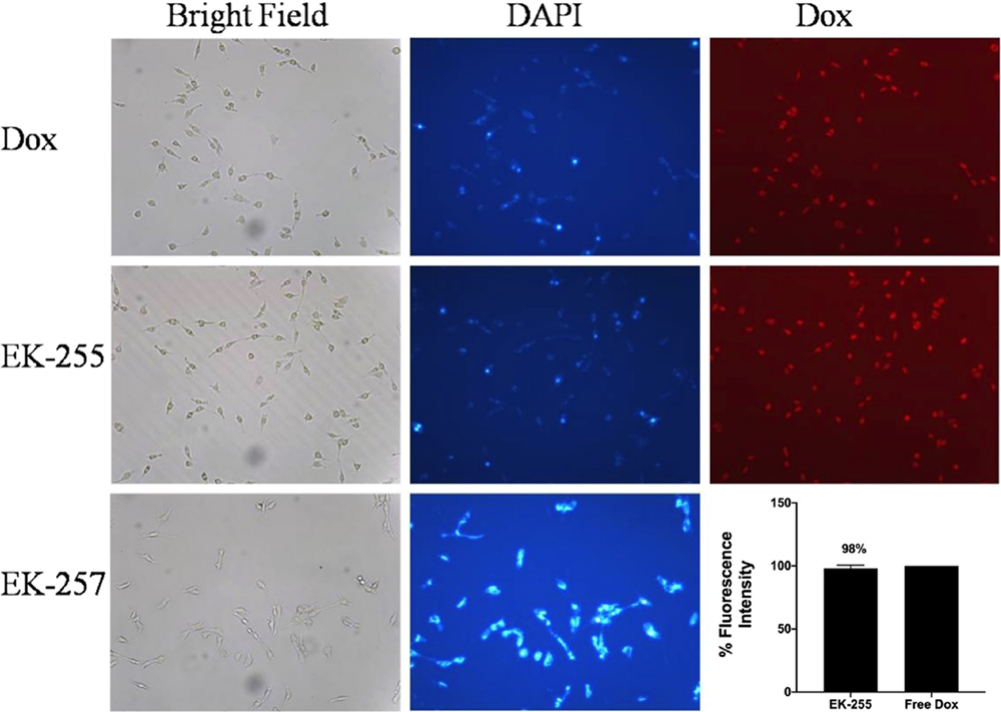
Cellular uptake analysis by fluorescence microscopy. Microscopic images were taken at 40× magnification. Histogram shows the quantitative analysis of cellular uptake.

Cellular uptake of DOX was assessed by microscopic evaluation of cells when treated with DOX and the micelles. Cellular uptake of free DOX was determined around 100%, while the uptake was approximately 98% when the cells were treated with DOX-loaded micelles (EK-255) for 4 h (Figure 9). The results showed that the micelles with DOX can successfully release the DOX content within 4 h and the micelles without DOX (EK257) did not induce any cell death within this period. Therefore, we propose that the micellar structure developed is a successful targeted drug delivery system with no cellular toxicity.

### Annexin V Staining Assay

**Figure 10:**
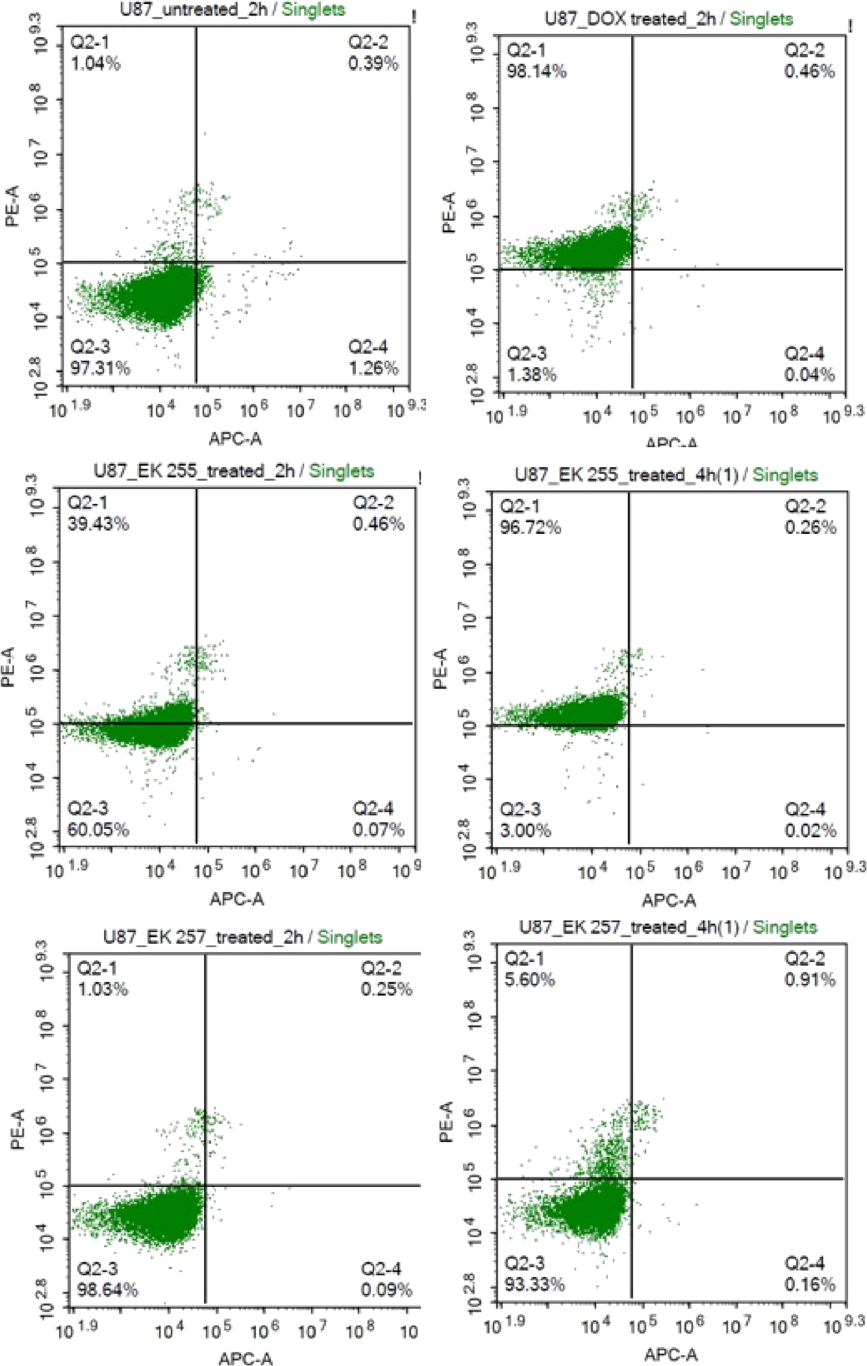
Annexin V analysis by flow cytometry. Cell death effects of free DOX, DOX-loaded micelle (EK-255), and empty micelle (EK-257) in U87-MG cells.

Annexin V staining was used to assess the extent of cell death with the percentage of early, late apoptosis, and death cells. Untreated U87-MG cells showed no cell death or apoptosis while 2 h of DOX treated cells showed 98% of dead cells. The cells treated with DOX-loaded micelle (EK255) for 2 h showed 39% of cell death, and those treated for 4 h of treatment caused 97% of cells to die which is consistent with free DOX treatment. However, neither 2 h nor 4 h of empty micelle (EK257) treatment caused significant cell death (Figure 10). The results indicated that drug release from the DOX-loaded micelles occurs within 4 h of treatment. DOX caused apoptosis was not detected in both 2 and 4 h of treatments. Hence, we can speculate that the effect of DOX is immediate and occurs in less than 2 h.

## Conclusions

In conclusion, a micellar drug carrier was fabricated from P(MAG-*co*-HEMA)-*b*-PBAE to realize pH-responsive release and potentially active targeting cancer cells. The P(MAG-*co*-HEMA) block was chosen to be hydrophilic and a cancer cell targeting block with glucose groups, while PBAE was chosen as a pH-sensitive hydrophobic and degradable segment. The amphiphilic polymer formed a micellar structure above p*K*_b_ (>6.75) and released the hydrophobic model drug DOX below p*K*_b_ (<6.75). Drug delivery potential was evaluated by cell viability assays for both the noncancerous HUVEC cell line and glioblastoma cell line U87-MG. While encapsulated DOX into the polymeric micelles was not toxic in noncancerous HUVEC cells, being toxic only to cancer cells indicates that it is a potential specific cell targeting strategy in the treatment of cancer. Our results are promising for future in vivo studies.

## Supporting Information Available

Reaction schemes, NMR spectra, FTIR spectra, and GPC chromatograms (DOCX)
