# Supplementary material for: Glycopolymer and Poly(β-amino ester)-Based Amphiphilic Block Copolymer as a Drug Carrier
Source: Biomacromolecules. 2022 Nov 1;23(11):4896–908. doi: 10.1021/acs.biomac.2c01076 (PMC9667500; doi:10.1021/acs.biomac.2c01076)
Supplement: Supplementary file 1 — bm2c01076_si_001.docx [file bm2c01076_si_001.docx]

**Supporting Information**

Glycopolymer and Poly(β-amino ester) Based Amphiphilic Block Copolymer as a Drug Carrier

Elif L. Sahkulubey Kahveci^⁑^, Muhammet U. Kahveci^‡^, Asuman Celebi^⁂^, Timucin Avsar^⁂^, Serap Derman^⁑^

^⁑^Yildiz Technical University, Faculty of Chemical and Metallurgical Engineering, Department of Bioengineering, Davutpasa Campus, Esenler, 34210 Istanbul, Turkey

^‡^ Istanbul Technical University, Faculty of Science and Letters, Department of Chemistry, Maslak, Sariyer, 34467 Istanbul, Turkey

^⁂^Department of Medical Biology, School of Medicine, Bahcesehir University, Goztepe, 34734 Istanbul, Turkey

E-mail: [kahvecimuh@itu.edu.tr](mailto:kahvecimuh@itu.edu.tr); [serapacar5@gmail.com](mailto:serapacar5@gmail.com)

**Scheme S1.** Synthesis of bis-acrylate functional PBAE by Michael Addition.

**Scheme S2.** Synthesis of Norbornene functional PBAE (NB-PBAE-NB)

**Scheme S3.** Synthesis of 2-deoxy-2-methacrylamido glucopyranose (MAG)

**Scheme S4.** Synthesis of Poly[(2-deoxy-2-methacrylamido glucopyranose)-*co*-(2-hydroxyethyl methacrylate)] P(MAG-*co*-HEMA)

**Scheme S5.** Synthesis of Poly[(2-deoxy-2-methacrylamido glucopyranose)-*co*-(2-hydroxyethyl methacrylate)-Tz] P(MAG-*co*-HEMA)-Tz

**Scheme S5.** Synthesis of poly(2-deoxy-2-methacrylamido-D-glucose-*co*-2-hydroxyethyl methacrylate)-*b*-poly(β-amino ester) [P(MAG-*co*-HEMA)-*b*-PBAE] by tetrazine click.

**
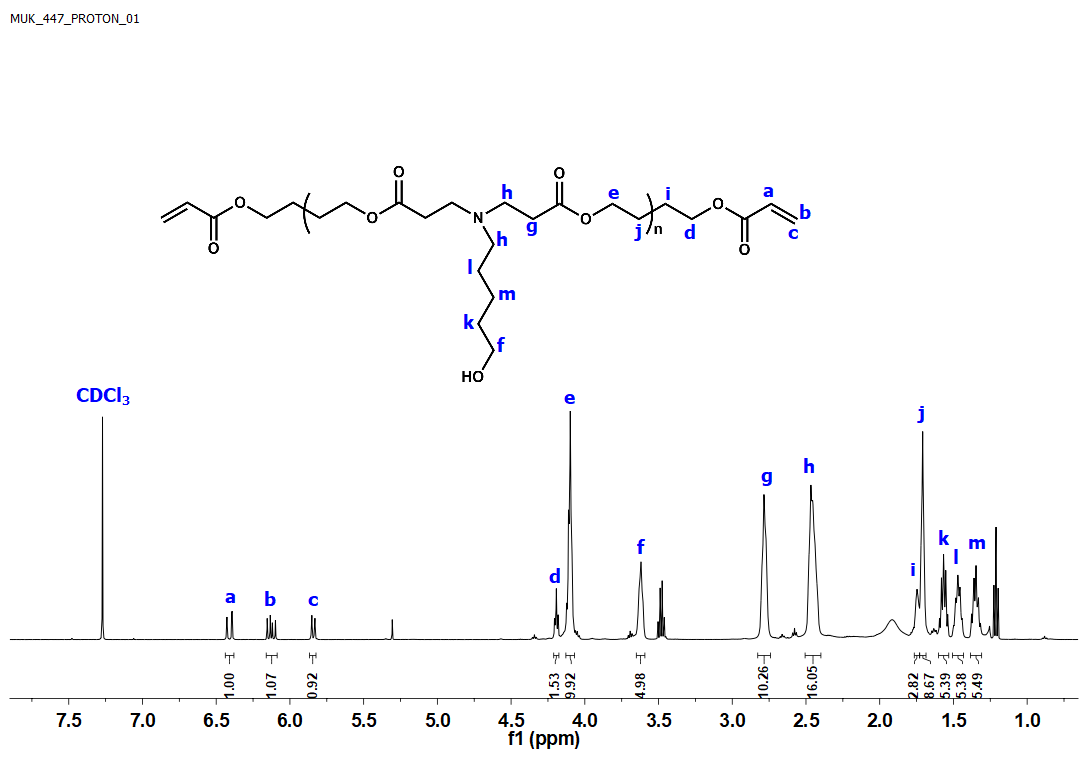
**

**Figure S1.** ^1^H-NMR spectrum of PBAE diacrylate in CDCl_3_.

**
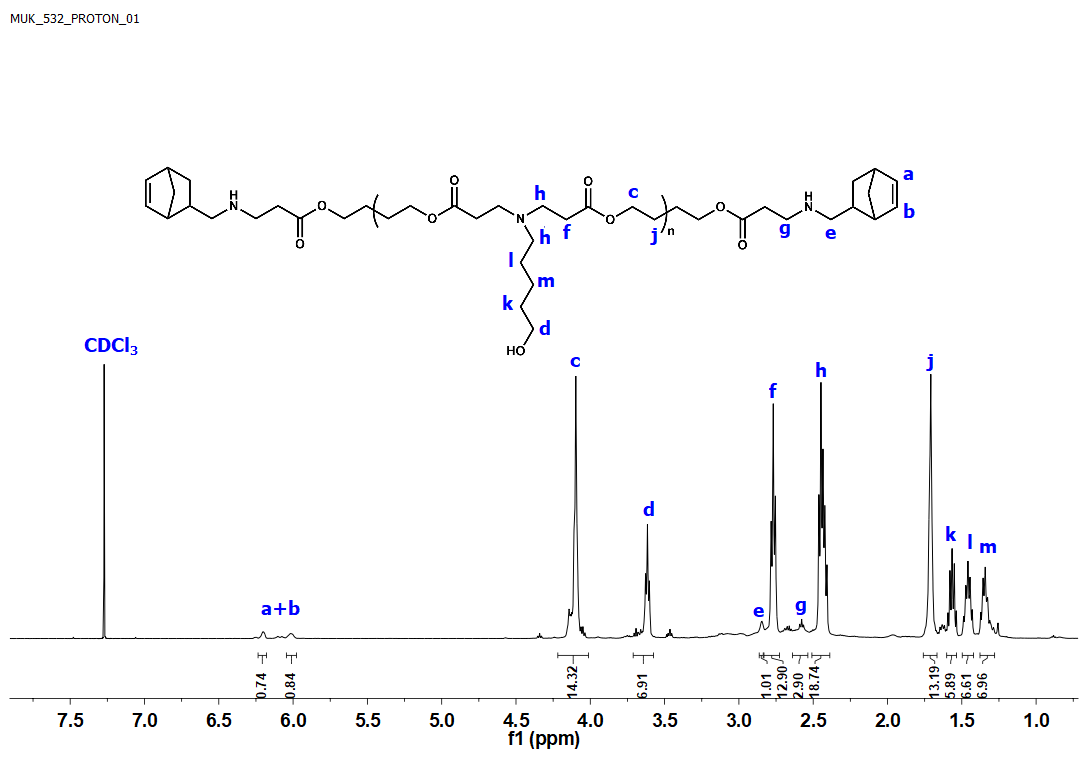

Figure S2.** ^1^H-NMR spectrum of norbornene functional PBAE (NB-PBAE-NB) in CDCl_3_.

**

**

**Figure S3.** FTIR spectra of PBAE diacrylate and norbornene functional PBAE (NB-PBAE-NB).

**
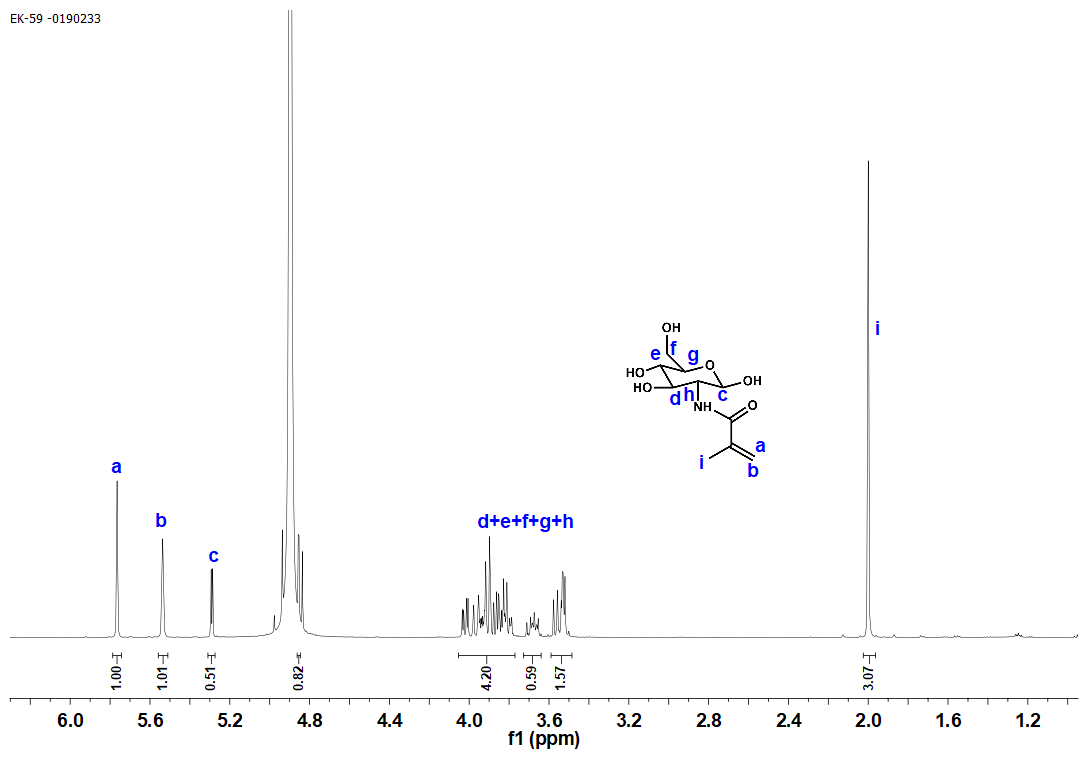
**

**Figure S4.** ^1^H-NMR spectrum of 2-deoxy-2-methacrylamido-D-glucose (MAG) in D_2_O.

**
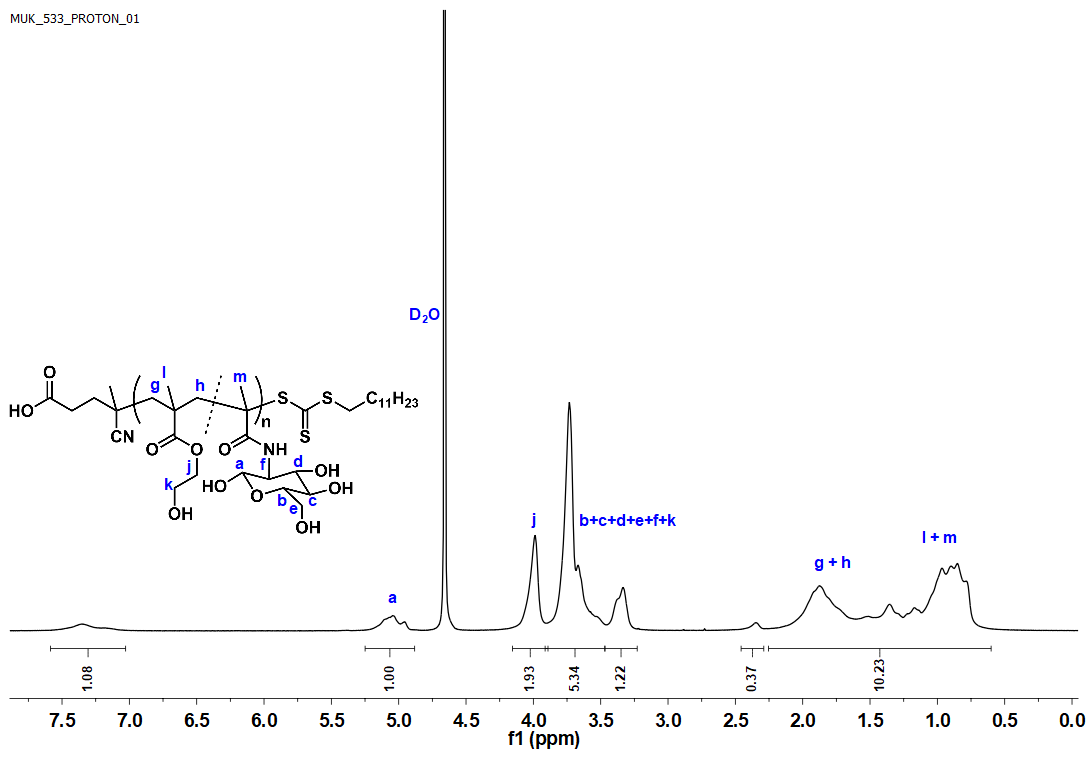
**

**Figure S5.** ^1^H-NMR spectrum of P(MAG-*co*-HEMA) in D_2_O.

**
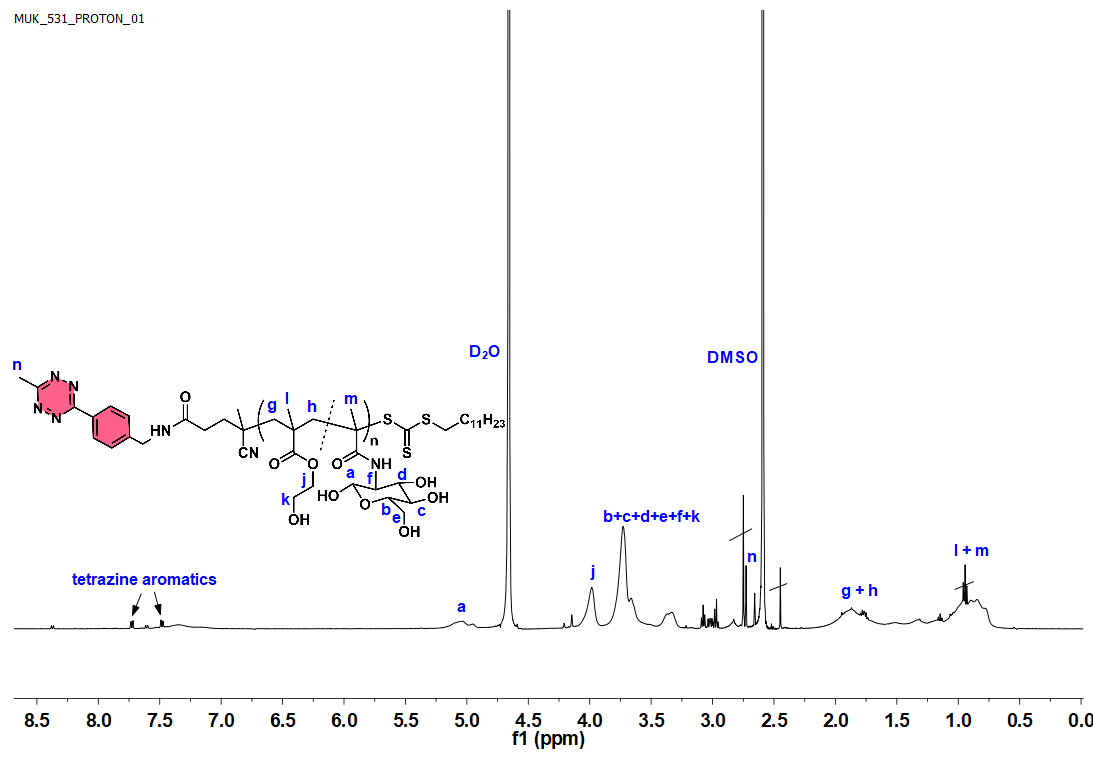
**

**
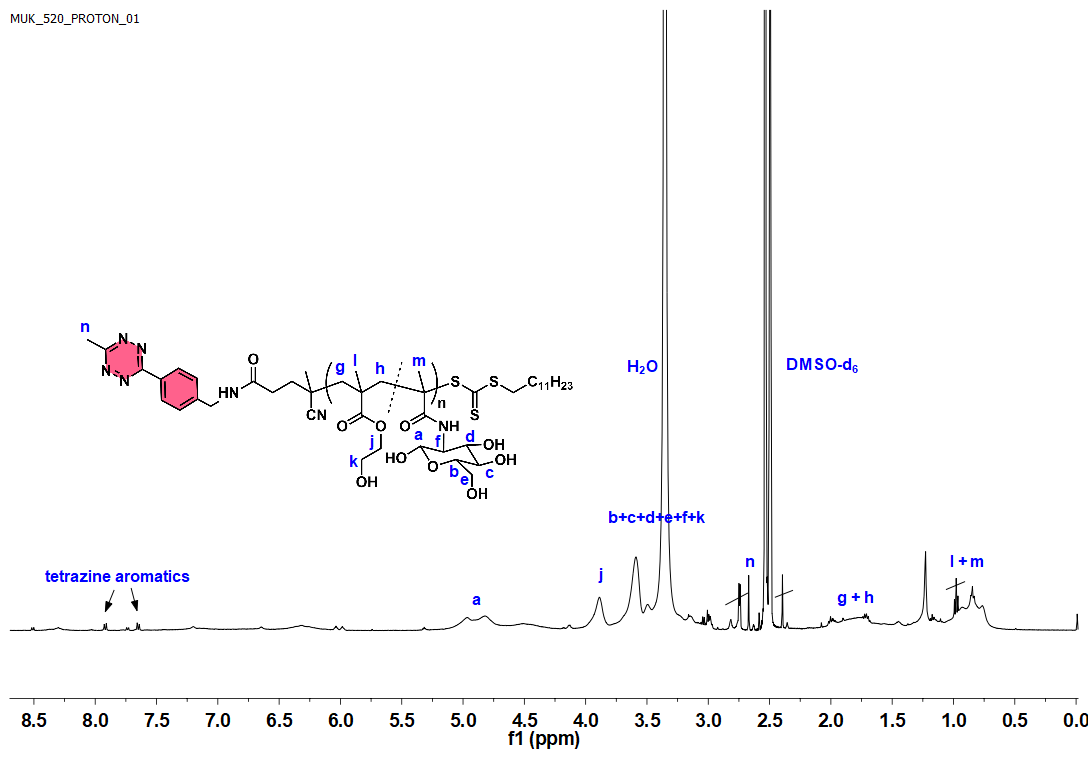
**

**Figure S6.** ^1^H-NMR spectrum of P(MAG-*co*-HEMA)-Tz in D_2_O and DMSO-*d*_6_.

**

**

**Figure S7.** FTIR spectra of P(MAG-*co*-HEMA) and P(MAG-*co*-HEMA)-Tz.

**
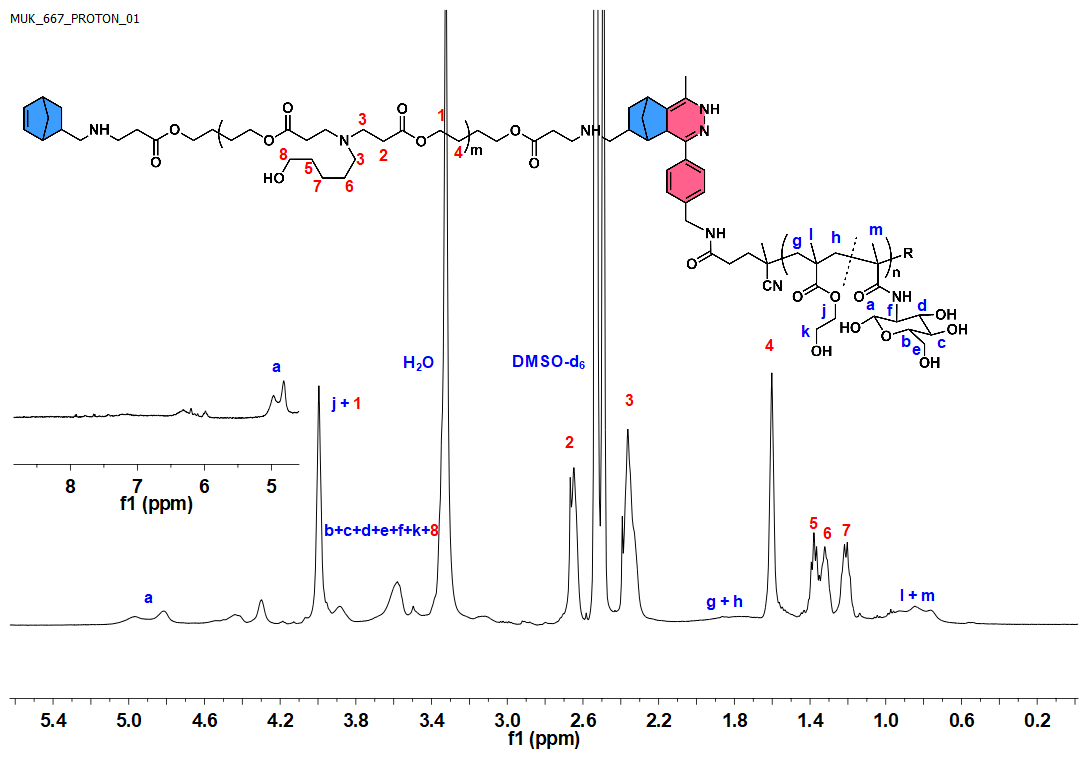
**

**Figure S8.** ^1^H-NMR spectrum of P(MAG-*co*-HEMA)-*b*-PBAE in DMSO-*d*_6_.







**Figure S9.** GPC chromatograms of P(MAG-*co*-HEMA) (A) and P(MAG-*co*-HEMA)-*b*-PBAE (B) (Left: refractive index signal; right: light scattering signal).
